# External Lumbar Drainage for Refractory Intracranial Hypertension in Acute Brain Injury: A Narrative Review and Case Series

**DOI:** 10.7759/cureus.116319

**Published:** 2026-09-15

**Authors:** Teresa Maria Caires Gomes da Silva, Matilde Drumond, Margarida Fernandes, Susana Afonso, Ary Sousa, José Jácome, Luís Morais, Bruno Maia

**Affiliations:** 1 Department of Critical Care Medicine, Hospital Dr. Nélio Mendonça, Funchal, PRT; 2 Department of Critical Care Medicine, Unidade Local de Saúde de São José, Lisbon, PRT

**Keywords:** acute brain injury, cerebrospinal fluid, external lumbar drainage, intracranial hypertension, intracranial pressure, neuromonitoring, traumatic brain injury

## Abstract

Refractory intracranial hypertension in the setting of acute brain injury (ABI) is defined as sustained intracranial pressure (ICP) elevation above 22 mmHg despite escalating medical management. When unresponsive to these therapies, mortality reaches 84%-100%. External lumbar drainage (ELD) has emerged as a minimally invasive adjunct for cerebrospinal fluid (CSF) diversion, either as complementary to or alternative to external ventricular drainage (EVD), potentially avoiding the need for barbiturate coma or decompressive craniectomy. The objective of this paper is to review current evidence on the use of ELD in refractory intracranial hypertension following ABI and to present a single-center case series illustrating its clinical application, efficacy, and safety. We conducted a narrative review of the literature and retrospectively analyzed five consecutive patients admitted to a neurointensive care unit between August 2025 and April 2026 who were treated with ELD for refractory intracranial hypertension. Patient demographics, clinical severity scores (Glasgow Coma Scale (GCS), Simplified Acute Physiology Score 3 (SAPS 3), Sequential Organ Failure Assessment (SOFA)), ICP values, timing of ELD placement, treatment escalation, length of stay, mechanical ventilation days, destination at discharge, and 28-day Glasgow Outcome Scale (GOS) were recorded. Five patients (four male; mean age 38.4 years) were treated with ELD: three with traumatic brain injury (TBI), one with hypertensive intracerebral hemorrhage, and one with aneurysmal subarachnoid hemorrhage (aSAH). The median time to ELD placement was three days (range 2-8). In two patients, ELD was implemented as a tier 2 measure following sedation and analgesia, osmotherapy, and head-of-bed elevation. In the remaining patients, ELD was used as a tier 3 intervention, combined with decompressive craniectomy (n = 2) and/or barbiturate coma (n = 2). CSF drainage was guided by ICP elevation, performed only when it exceeded 20 mmHg; however, three of the five patients required continuous rate-based drainage (5 mL/h in Cases 1 and 5 and 10 mL/h in Case 4) because of sustained intracranial hypertension. Sustained ICP reduction with a favorable neurological outcome (GOS 4) was observed in one patient treated with early ELD (second day). Three patients died (one of whom died following transfer to another institution), and one patient developed two episodes of over-drainage due to accidental system disconnection, requiring ELD removal after one day. No infectious complications were recorded. Our conclusion is that ELD is a feasible, minimally invasive therapeutic option for refractory intracranial hypertension in ABI. Early placement, appropriate patient selection based on neuroimaging (patent basal cisterns, absence of significant mass effect), and continuous neuromonitoring are critical for safety and efficacy. Prospective randomized trials are needed to define optimal timing, patient phenotype, and drainage protocols.

## Introduction

Acute brain injury (ABI) is a leading cause of death and disability worldwide. It can be due to traumatic brain injury (TBI), ischemic cerebral stroke, hypertensive intracerebral hemorrhage, or aneurysmal subarachnoid hemorrhage (aSAH). Severe TBI, defined by a Glasgow Coma Scale (GCS) [1] score of 8 or less at admission, is frequently complicated by intracranial hypertension - defined as sustained intracranial pressure (ICP) above 20-22 mmHg - which drives secondary neurological injury through impaired cerebral perfusion pressure (CPP) and cerebral ischemia [2].

The Monro-Kellie doctrine establishes that the intracranial compartment is a fixed-volume space containing brain parenchyma (~80%), blood (~10%), and cerebrospinal fluid (CSF) (~10%), with a normal ICP below 15 mmHg. Any increase in one compartment must be compensated by a decrease in another; when compensatory mechanisms are exhausted, ICP rises uncontrollably [3].

Recent advances in understanding the glymphatic system have reframed our conception of CSF dynamics in ABI. This brain-wide perivascular drainage network, dependent on aquaporin-4 (AQP4) water channels at astrocytic endfeet, is the primary mechanism for clearing metabolic waste products, including β-amyloid and tau proteins, from the brain parenchyma [4,5]. Across the spectrum of ABI, this system is critically disrupted, though by distinct mechanisms: in TBI, mechanical forces cause loss of AQP4 polarization and impaired CSF-interstitial fluid exchange, compounded by microglial inflammation [4,6]; in aSAH, fibrin deposits within perivascular spaces directly obstruct glymphatic flow, impairing clearance of neurotoxic blood components and contributing to post-hemorrhagic hydrocephalus [5,6]; and in ischemic stroke, AQP4 depolarization and impaired parenchymal penetration predominate in the subacute phase [4,5]. The resulting accumulation of toxic metabolites and amplified neuroinflammation compound secondary brain injury and may accelerate long-term neurodegeneration [4-6]. Restoration of CSF circulation through diversion strategies - including external lumbar drainage (ELD) - may therefore exert beneficial effects beyond simple volume reduction, potentially restoring glymphatic flow along perivascular pathways [6,7].

Across different forms of ABI, intracranial hypertension develops through distinct mechanisms. In ischemic stroke, cellular energy failure leads to cytotoxic edema, characterized by intracellular water accumulation due to disruption of ionic homeostasis [8]. In contrast, aSAH is associated with blood-brain barrier (BBB) disruption, neuroinflammation, and the development of vasogenic edema, resulting in extracellular fluid accumulation [9]. Moderate-to-severe TBI causes intracranial hypertension through two main mechanisms: (1) diffuse cytotoxic cerebral edema (due to cellular energy failure) and (2) focal mass effect from intracranial hemorrhage or contusion. Both mechanisms compress the remaining CSF and venous compartments, eroding compensatory reserve [10]. Regardless of the underlying etiology, the progressive accumulation of edema and mass effect exhausts intracranial compensatory mechanisms, causing elevated ICP, reduced CPP, cerebral ischemia, and ultimately a self-propagating cycle of secondary brain injury [2].

Current management in TBI follows a tiered, stepwise approach as outlined by the Seattle International Severe Traumatic Brain Injury Consensus Conference (SIBICC) protocol. Tier 1 interventions include sedoanalgesia, normothermia, normoglycemia, osmotherapy (mannitol or hypertonic saline), CPP optimization, and CSF drainage via external ventricular drainage (EVD) when in situ. Tier 2 escalates to mild controlled hypocapnia (PaCO₂ 32-35 mmHg), neuromuscular blockade, and mean arterial pressure (MAP) challenge. Tier 3 - reserved for truly refractory ICP - includes barbiturate coma and decompressive craniectomy, both carrying significant morbidity: hemodynamic instability, prolonged awakening, immunosuppression, surgery-related infections, and the need for subsequent cranioplasty [2,11].

ELD offers a potentially valuable intermediate option - less invasive than EVD or craniectomy - by draining CSF from the lumbar subarachnoid space, thereby reducing intracranial CSF volume and ICP. The evidence supporting ELD in TBI remains limited and heterogeneous. While multiple studies demonstrate consistent short-term ICP reduction, its effect on long-term neurological outcome is uncertain [7,12,13]. Nevertheless, emerging data suggest that early ELD, combined with appropriate patient selection based on neuroimaging, may improve outcomes and reduce the need for tier 2 and tier 3 escalation [13-15].

In practice, most reported protocols favor controlled, rate-limited CSF removal in the range of 5-10 mL/h rather than high-volume free drainage [7,14,16,17]. This contrasts with EVD, where continuous CSF diversion occurs under direct ICP monitoring at a fixed anatomical reference (external auditory meatus or foramen of Monro). A key safety consideration specific to ELD is the potential for a craniospinal pressure gradient to develop between the ventricular and lumbar compartments, particularly when both EVD and ELD are used concomitantly. Early recognition of this gradient - historically termed the lumboventricular pressure gradient - has been proposed as a marker of impending over-drainage and herniation risk in patients with intracranial hypertension of hemorrhagic or traumatic origin [18]. Building on this principle, subsequent TBI-specific series have incorporated structured gradient monitoring and stepwise adjustment of the lumbar drainage level relative to the foramen of Monro to titrate ICP targets safely [16], while dedicated scoring systems have been proposed to formalize the assessment of herniation risk before and during lumbar drainage in refractory post-traumatic intracranial hypertension [19]. Consistent with this evidence, our institutional protocol mandates interruption of lumbar drainage whenever the ventricular-lumbar pressure difference exceeds 5 mmHg, in either direction.

While ELD is increasingly proposed as an intermediate option in the SIBICC tiered framework for refractory intracranial hypertension, evidence guiding its safe and effective use - including patient selection, gradient monitoring, and drainage rate - remains limited to small, heterogeneous series. This case series aims to describe our single-center experience with ELD, including gradient-based monitoring practices, in five patients with refractory intracranial hypertension of diverse etiologies.

## Case presentation

We retrospectively analyzed five consecutive patients admitted to a neurointensive care unit between August 2025 and April 2026 who received ELD for refractory intracranial hypertension. Data were extracted from the electronic medical record. Patient confidentiality was maintained in accordance with applicable regulations. This case series was not reported following the CARE (CAse REport) guidelines [20].

Demographic variables, admission GCS [1], Simplified Acute Physiology Score 3 (SAPS 3) [21], Sequential Organ Failure Assessment (SOFA) [22] score at admission and at discharge, timing of ICP sensor placement, admission ICP, timing of ELD insertion, ELD drainage protocol, co-interventions (barbiturate coma, decompressive craniectomy), hospital length of stay, days of mechanical ventilation, GCS at discharge, and Glasgow Outcome Scale (GOS) [23] at 28 days were recorded.

ICP values were recorded using an internal electronic system in each patient's database. For each day, the highest sustained ICP value documented in the clinical record and considered clinically relevant by the treating physician was recorded. ICP spikes related to nursing care, mobilization, or transient stimuli were excluded. Neuroimaging was classified according to the CSF reserve grading scale proposed by Dagod et al. [16], which stratifies patients from Grade 1 (physiologic supratentorial CSF reserve, favorable for early ELD) to Grade 7 (diffuse brain swelling with complete loss of supratentorial and infratentorial CSF reserve, absolute contraindication to ELD).

Patient selection and neuroimaging-based contraindications

The main risk of ELD is tonsillar herniation due to a pressure-gradient reversal caused by over-drainage. Dagod et al. proposed a 7-grade classification of intracranial CSF reserve based on computed tomography (CT) findings: Grades 1-3 (physiologic to infratentorial-only reserve) are favorable for early ELD placement (ICP target 10-15 mmHg); Grade 4 (small extra-axial lesion, midline shift < 5 mm) warrants late ELD with higher ICP target (15-22 mmHg); Grade 5 (focal intraparenchymal lesion or midline shift > 5 mm) suggests medical treatment priority with ELD only as a rescue measure at ICP > 30 mmHg; Grades 6-7 represent absolute contraindications [16].

Patient characteristics

Five patients received ELD during the study period (August 2025-April 2026): four men and one woman. The mean age was 38.4 years (range 20-57). Causes of admission included TBI (n = 3), hypertensive intracerebral hemorrhage (n = 1), and aSAH (n = 1). All patients required mechanical ventilation. Mean SAPS 3 was 62.6, and mean SOFA at admission was 9.8 (Table 1).

**Table 1:** Patient characteristics, clinical course, and outcomes. aSAH: aneurysmal subarachnoid hemorrhage; D: day; DC: discharge; ELD: external lumbar drain; GCS: Glasgow Coma Scale; GOS: Glasgow Outcome Scale; Hyp ICH: hypertensive intracerebral hemorrhage; ICP: intracranial pressure (mmHg); LOS: length of stay (days); MV: mechanical ventilation; SAPS 3: Simplified Acute Physiology Score 3; SOFA: Sequential Organ Failure Assessment; TBI: traumatic brain injury

Sex/age	Date	Cause	GCS D0	ICP (day)	ICP (mmHg)	ELD day	LOS (d)	MV (d)	SAPS 3	SOFA D0	SOFA DC	GCS DC	GOS D28
Case 1 M/20	Aug 2025	TBI	4	D1	15	D2	3	3	67	14	16	4	1
Case 2 M/43	Oct 2025	TBI	3	D1	21	D3	26	26	77	12	5	4	1
Case 3 M/37	Oct 2025	TBI	13	D1	50	D3	14	10	55	5	2	14	4
Case 4 M/35	Apr 2026	Hyp ICH	3	D1	43	D8	10	10	55	8	10	3	1
Case 5 F/57	Jan 2026	aSAH	15	D3	8	D6	22	15	59	10	3	7	3

Case series

Case 1: M/20, TBI (Aug 2025)

A 20-year-old male patient was admitted following severe polytrauma with an admission GCS of 4. ICP monitoring was placed on the first day with an initial ICP of 15 mmHg. Neuroimaging showed a CSF reserve Grade 1-2, compatible with early ELD placement. ELD was inserted on D2. Initially, the drainage strategy was ICP-targeted, with CSF drainage performed only when ICP exceeded 20 mmHg. However, because of sustained intracranial hypertension, the patient was subsequently managed with continuous drainage at 5 mL/h. Despite this intervention, ICP continued to rise. Anti-edematous measures, including sedoanalgesia, osmotherapy, mild hypocapnia, and MAP challenge, were instituted, followed by barbiturate coma on D3. ICP rose steadily. Despite all anti-edematous therapeutic measures, the patient was initially admitted with high severity scores due to primary injury (SAPS 3 67, SOFA 14). The extension of brain injury led to his death on D3 (GOS 1 on the 28th day) (Figures 1, 2).

**Figure 1:**
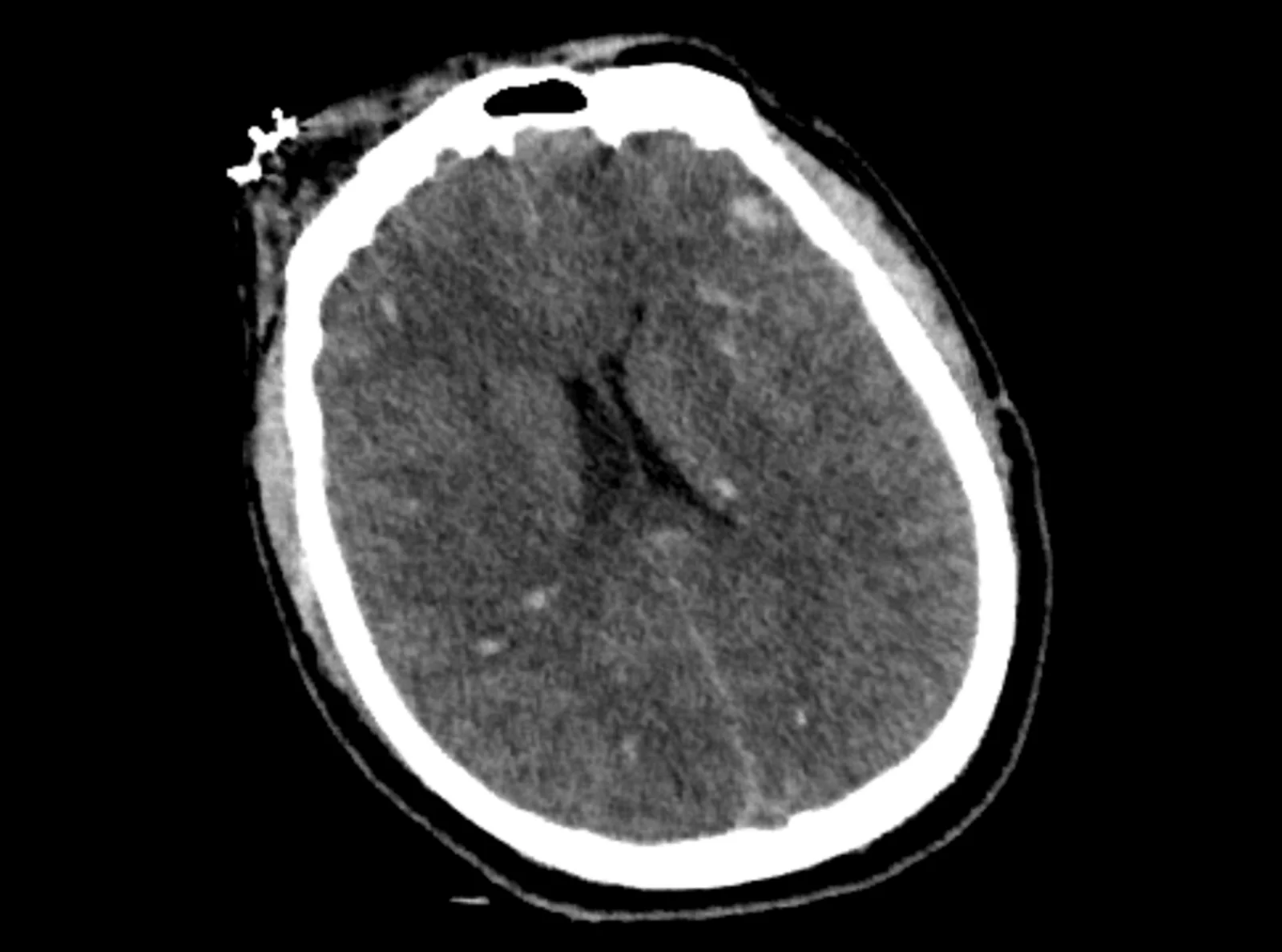
Non-contrast CT scan showing mild cerebral edema with effacement of sulci and bilateral hyperdense foci. CT: computed tomography

**Figure 2:**
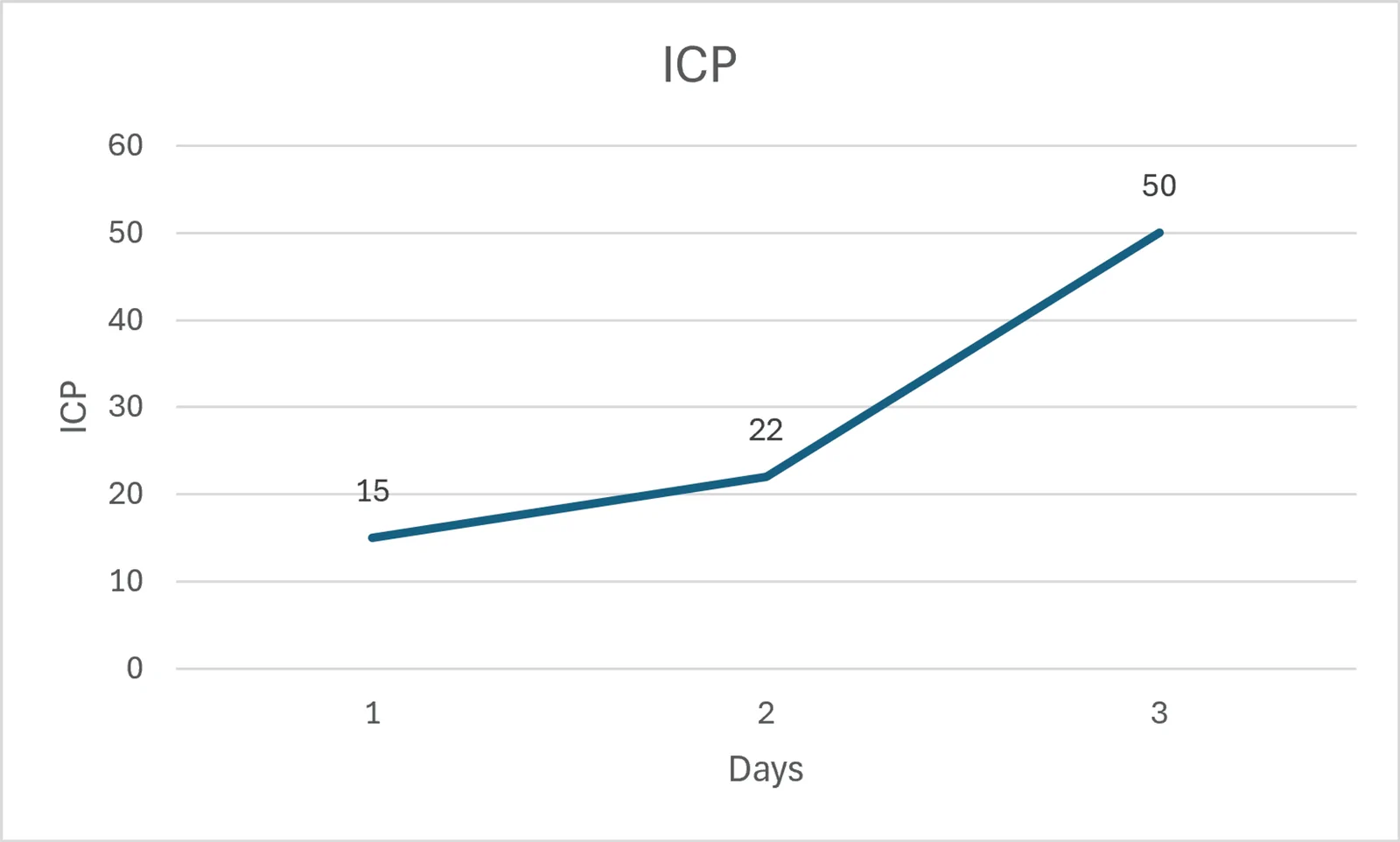
ICP evolution over time: Case 1: M/20 ICP: intracranial pressure (mmHg)

Case 2: M/43, TBI (Oct 2025)

A 43-year-old man with severe TBI was admitted with a GCS of 3 and an initial ICP of 21 mmHg. CT imaging demonstrated diffuse brain swelling classified as Grade 7 on the CSF reserve scale, reflecting heightened procedural risk for ELD. Sedoanalgesia and osmotherapy were initiated immediately; however, ICP continued to rise, prompting the induction of a barbiturate coma on D2. Despite maximal medical therapy, refractory intracranial hypertension persisted. Given the absence of remaining tier 3 alternatives, ELD was inserted as a last-resort rescue measure. The drain remained clamped and was opened intermittently only when ICP exceeded 20 mmHg. The patient was subsequently transferred to another institution and ultimately died, with a GOS 1 at 28 days. This case illustrates the challenging balance between imaging-based contraindications to ELD and the lack of effective alternative rescue therapies in refractory intracranial hypertension (Figures 3, 4).

**Figure 3:**
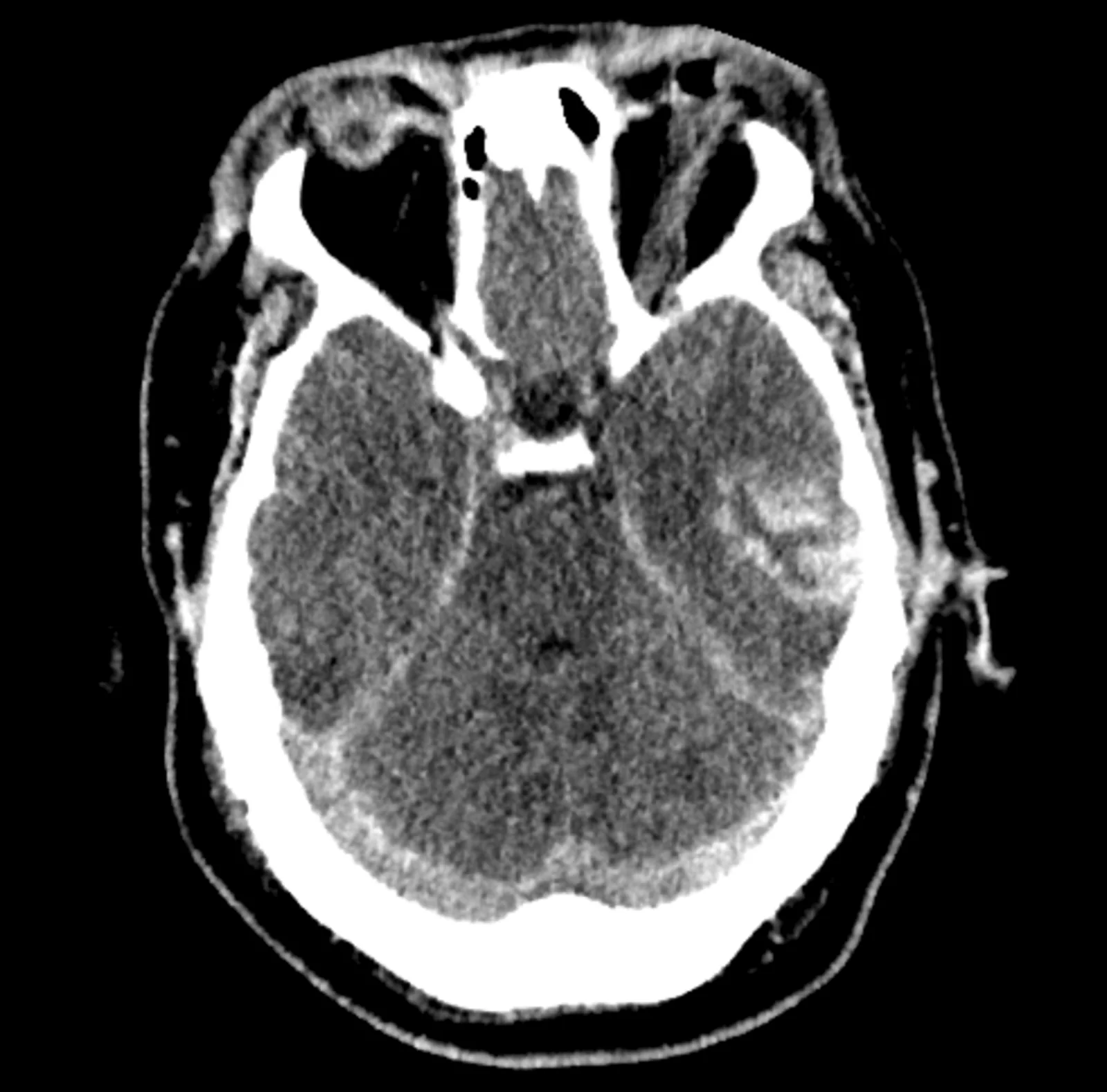
Non-contrast CT scan demonstrating diffuse edema with compression and effacement of the fourth ventricle. CT: computed tomography

**Figure 4:**
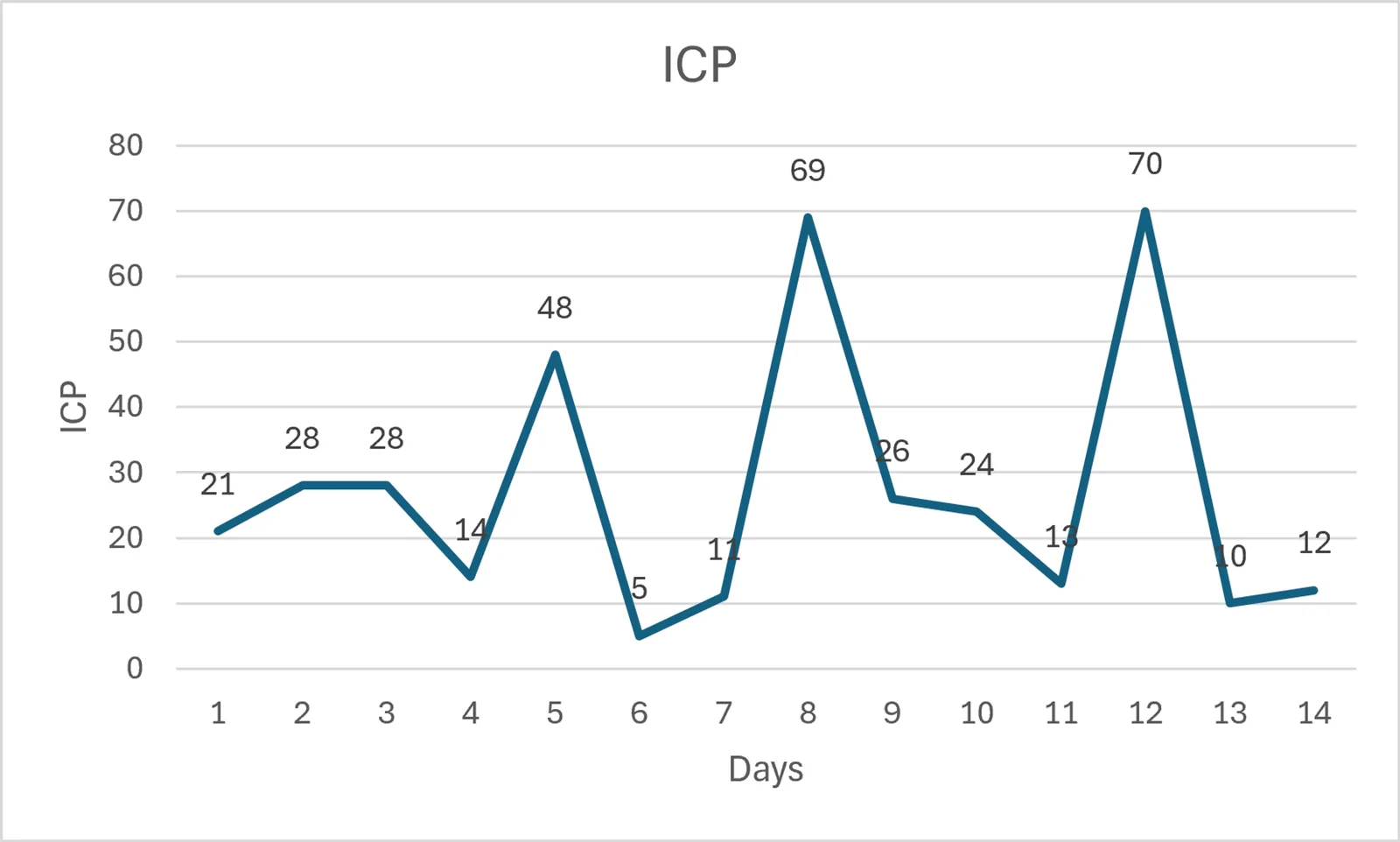
ICP evolution over time: Case 2: M/43 ICP: intracranial pressure (mmHg)

Case 3: M/37, TBI (Oct 2025)

A 37-year-old male patient with severe polytrauma was admitted with a GCS of 13 and a markedly elevated ICP of 50 mmHg on D1. CT corresponded to a Grade 1-2 on the CSF reserve scale. Anti-edematous measures, including sedoanalgesia, osmotherapy, and mild hypocapnia, were instituted, and after 48 hours, ELD was placed, targeting drainage only when ICP exceeded 20 mmHg. Following ELD insertion, accidental system disconnection resulted in two episodes of over-drainage, requiring ELD removal after one day, with no lasting clinical consequences for the patient. Despite this complication, ICP showed a progressive reduction pattern. The patient was discharged on D14 with GCS 14 and GOS 4 at 28 days - the best neurological outcome in the series - consistent with early ELD placement in a less severe anatomical substrate (Figures 5, 6).

**Figure 5:**
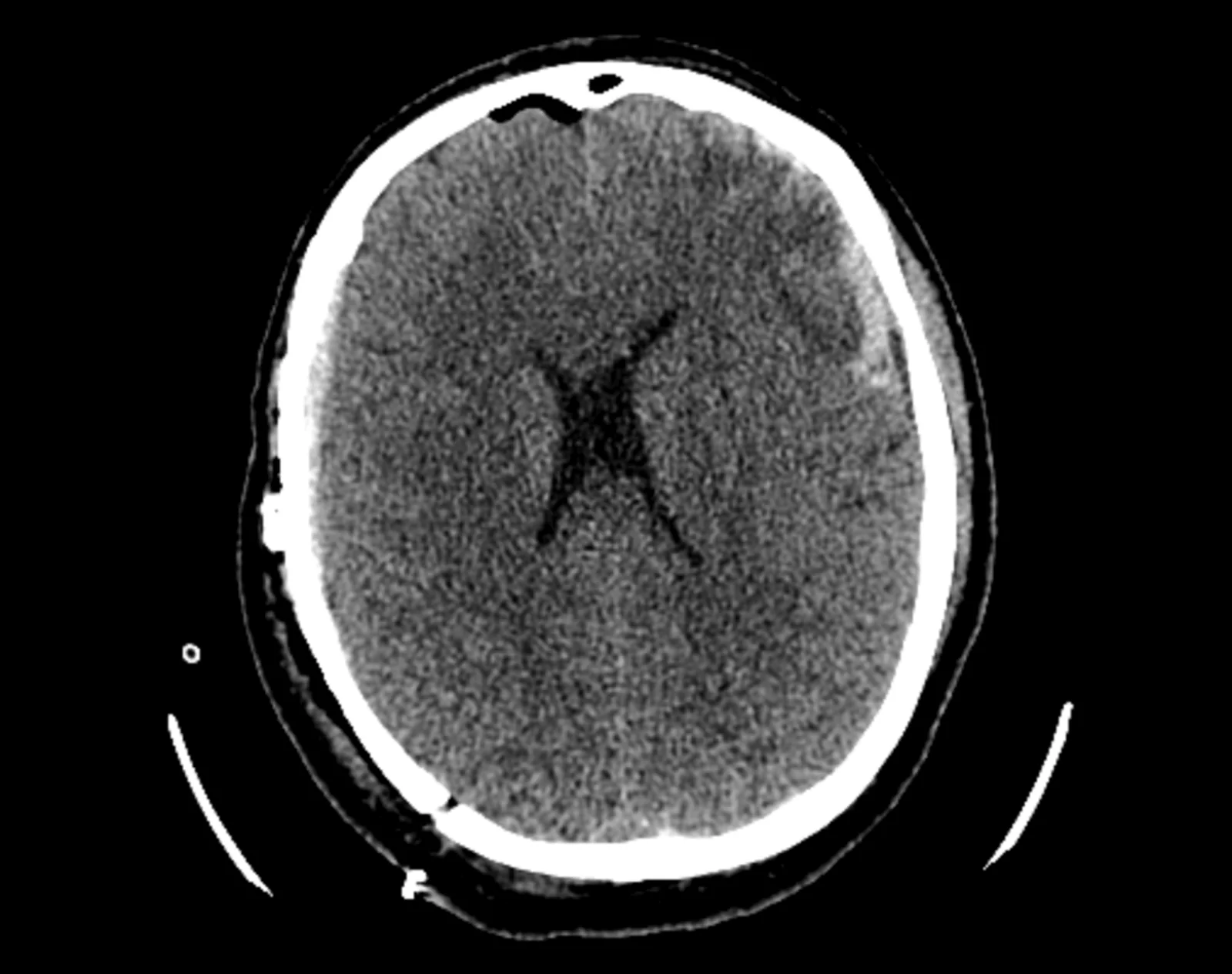
Non-contrast CT scan showing a hyperdense contusional focus in the frontal region with a surrounding hypodense edema halo. CT: computed tomography

**Figure 6:**
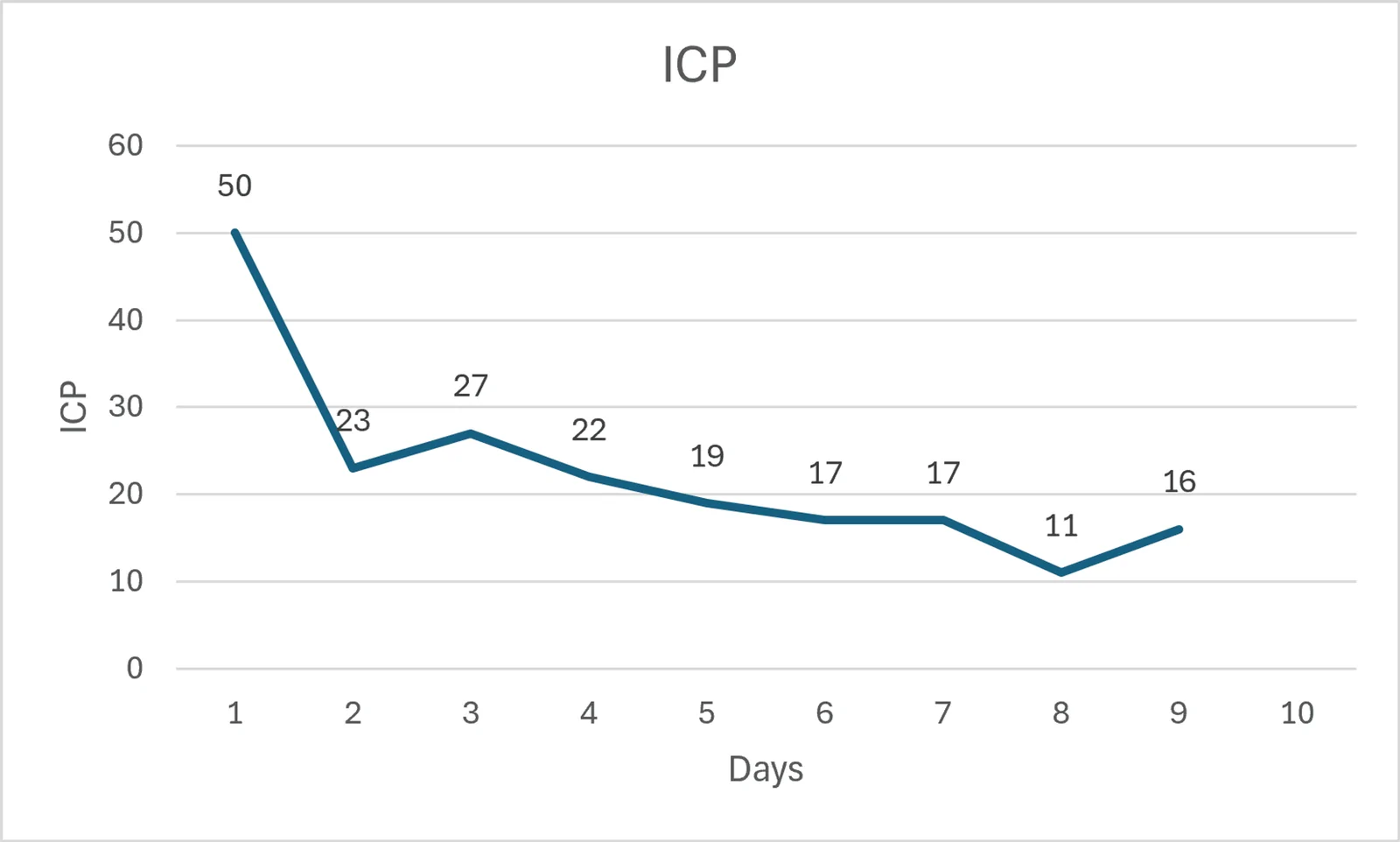
ICP evolution over time: Case 3: M/37 ICP: intracranial pressure (mmHg)

Case 4: M/35, Hypertensive Intracerebral Hemorrhage (Apr 2026)

A 35-year-old male patient with hypertensive intracerebral hemorrhage was admitted in a comatose state (GCS 3). ICP was 43 mmHg on D1. CT showed a focal intraparenchymal lesion with midline shift > 5 mm (Grade 5), indicating ELD only as a potential rescue measure at ICP > 30 mmHg with a high ICP target. Decompressive craniectomy was performed on D5. ELD was placed on D8 following persistent ICP elevation, draining at 10 mL/h. Despite all interventions, ICP remained elevated, and the patient died on D10 (GOS 1). This case illustrates the limited scope of rescue ELD in high-grade neuroimaging presentations (Figures 7, 8).

**Figure 7:**
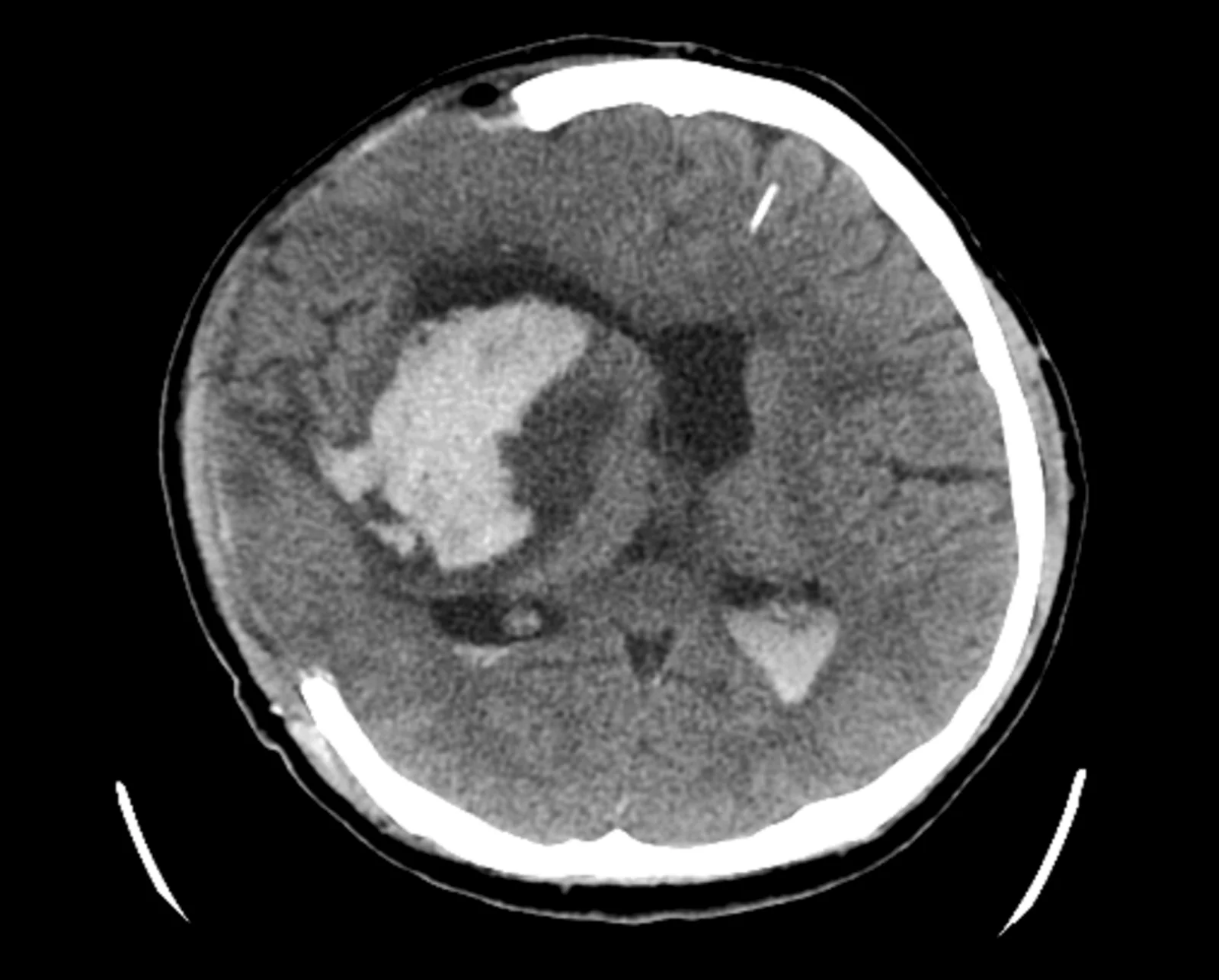
Non-contrast CT scan demonstrating a large hyperdense intraparenchymal hemorrhage in the right hemisphere with surrounding edema and midline shift. CT: computed tomography

**Figure 8:**
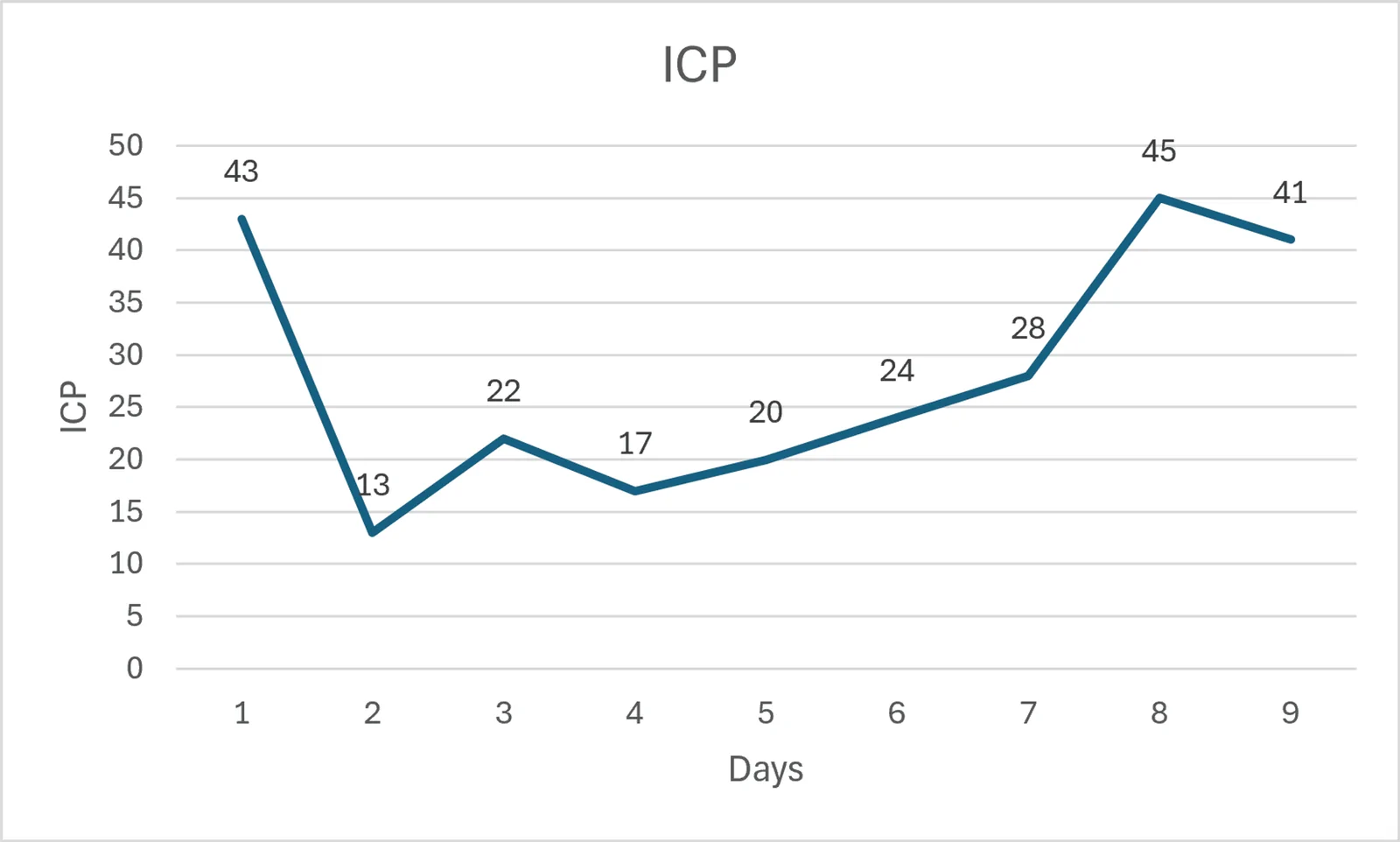
ICP evolution over time: Case 4: M/35 ICP: intracranial pressure (mmHg)

Case 5: F/57, aSAH (Jan 2026)

A 57-year-old woman with an aSAH was admitted with a GCS of 15. She underwent surgical clipping, and an ELD was placed postoperatively in accordance with institutional protocol for aSAH (modified Fisher 3-4, secured aneurysm, drainage initiated within 72 hours of rupture). This initial catheter was subsequently removed because of a technical malfunction. On D3, her neurological status deteriorated to a GCS of 7, prompting ICP monitoring. Although the worst ICP value on this day was 8 mmHg, it increased progressively over the following days. Brain CT revealed right hemispheric cerebral edema secondary to ischemic injury. First-tier therapies, including sedoanalgesia and osmotherapy, were initiated. Due to refractory intracranial hypertension, a decompressive craniectomy was performed on D5, followed by ELD placement on D6. CT findings corresponded to Grade 3-4 on the CSF reserve scale, supporting late ELD insertion with a higher ICP treatment threshold (15-22 mmHg). The drain was set to remove 5 mL/h of CSF. The patient was discharged with a GOS of 3 at 28 days (Figures 9, 10).

**Figure 9:**
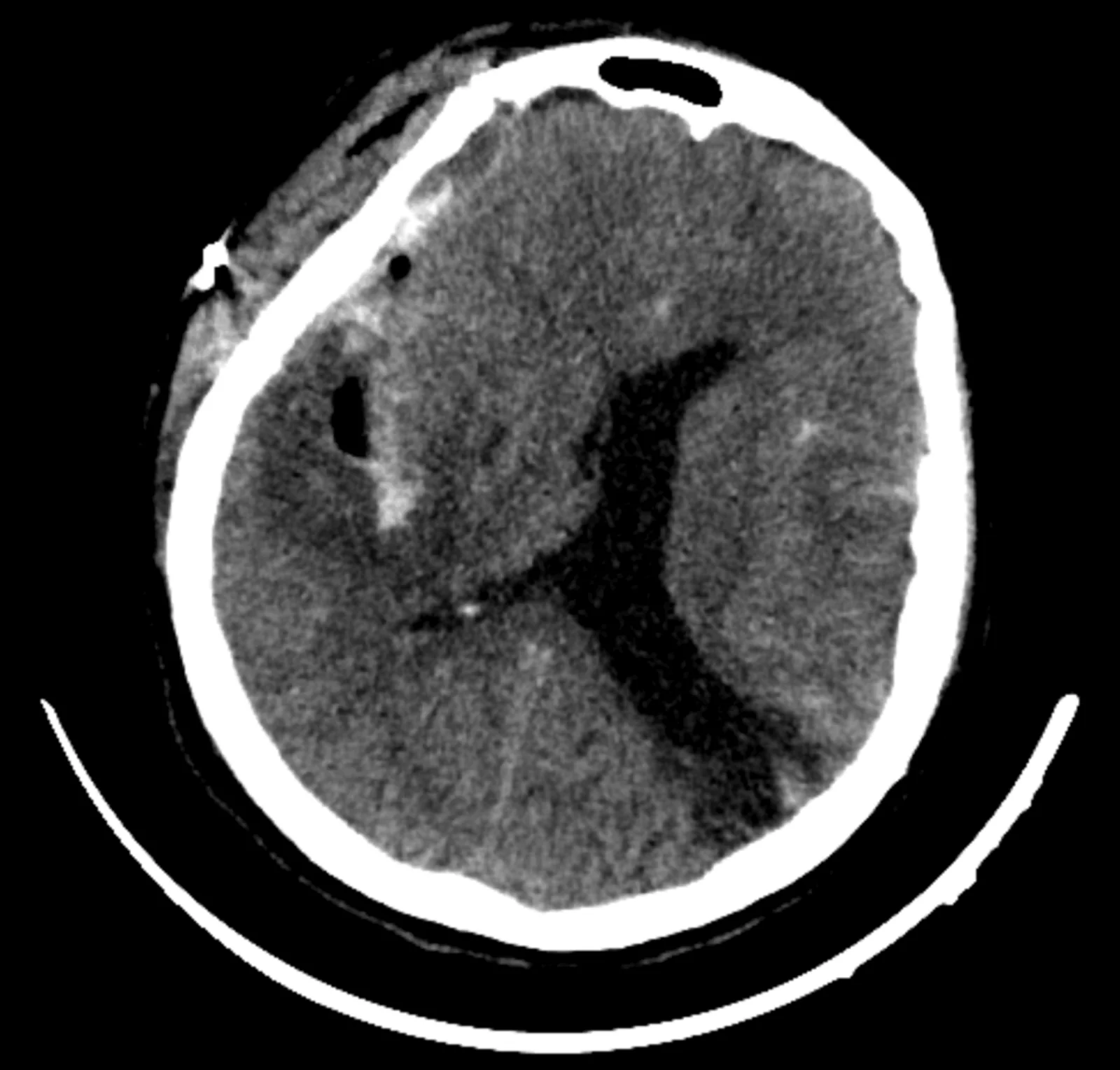
Post-operative non-contrast CT scan showing a small residual hyperdense collection with a discrete midline shift to the left. CT: computed tomography

**Figure 10:**
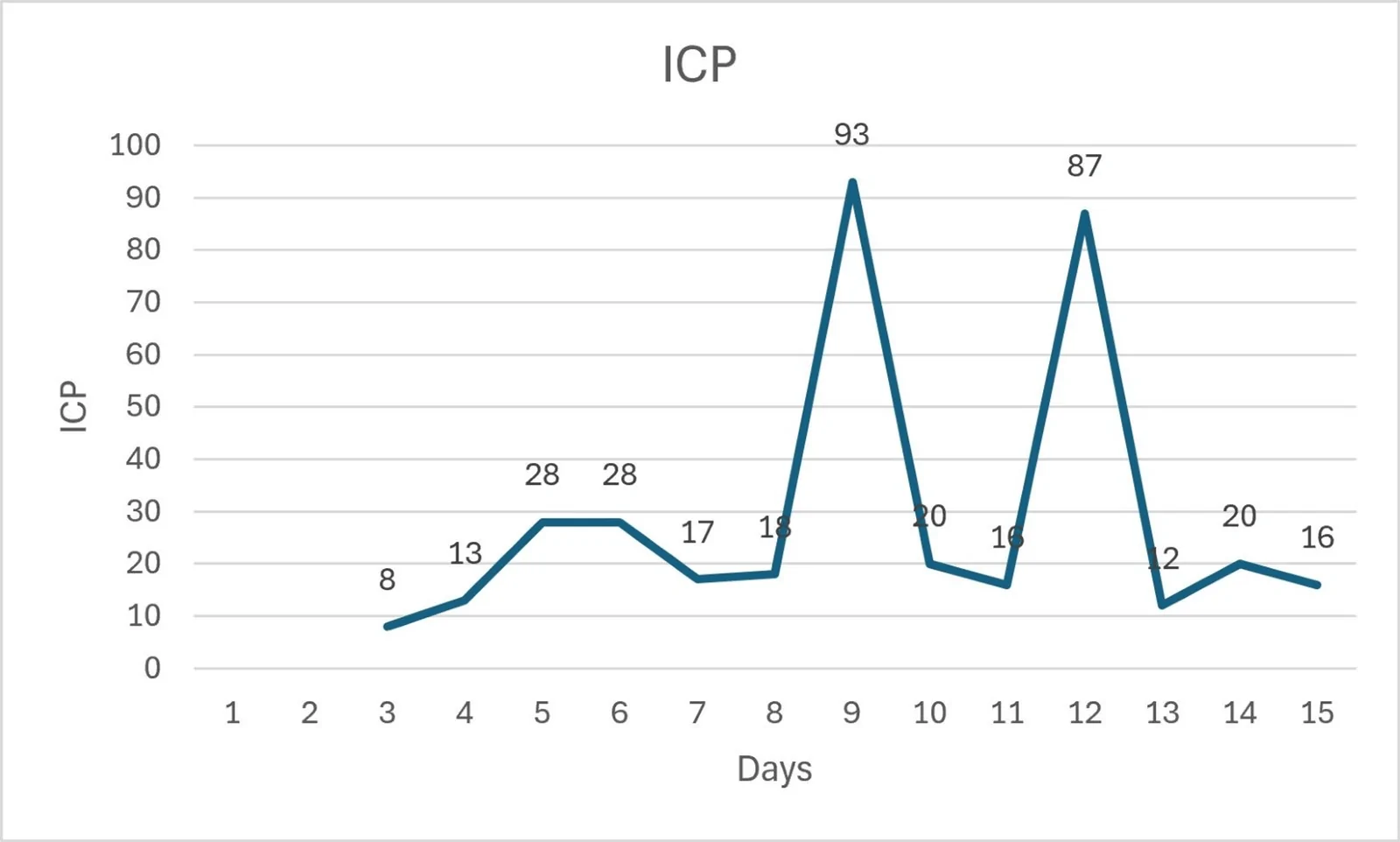
ICP evolution over time: Case 5: F/57 ICP: intracranial pressure (mmHg)

## Discussion

A narrative review was conducted by searching PubMed using the keywords "external lumbar drainage", "intracranial hypertension", and "acute brain injury". Studies published between 2001 and 2026 were included. Relevant prospective and retrospective studies, reviews, and case series were selected at the authors' discretion to summarize current evidence. Key findings are summarized in Table 2.

**Table 2:** Literature review of ELD in ABI. BTF: Brain Trauma Foundation; CPP: cerebral perfusion pressure; CT: computed tomography; ELD: external lumbar drainage; EVD: external ventricular drainage; GCS: Glasgow Coma Scale; GOS: Glasgow Outcome Scale; ICH: intracerebral hemorrhage; ICP: intracranial pressure; RCT: randomized controlled trial; SAH: subarachnoid hemorrhage; TBI: traumatic brain injury

Author (year)	Study type	Population	Methods/ELD protocol	Outcomes (ELD)
Abadal-Centellas et al. (2007) [7]	Prospective case series	n = 17; severe TBI with refractory ICP (tier 2 failure)	ELD instituted after failure of tier 2 therapies	Reduced mortality; improved functional outcome at 6 months
Ginalis et al. (2022) [24]	Literature review (11 studies)	Mixed; refractory TBI	Review of 6 retrospective + 5 prospective studies	Consistent ICP reduction; low complication rate. Mortality and functional outcome data are heterogeneous - no firm conclusions can be drawn
Soltész et al. (2024) [14]	Retrospective (2008-13) + prospective (2014-19)	n = 45; severe TBI	ELD placed within the first week, maintained ≥5 days	Sustained ICP reduction within 24 h; significantly reduced need for tier 2 and tier 3 interventions (p < 0.001); in-hospital mortality 16% vs. 48% in the general severe TBI population
Stevens et al. (2022) [12]	Systematic review (9 studies)	n = 230 (TBI, SAH, ICH)	ELD as adjunct therapy	Immediate and sustained ICP reduction; reduced medical therapy requirements. Insufficient data to draw firm conclusions on mortality benefit
Grille et al. (2024) [17]	Retrospective case series	n = 11; TBI, SAH, meningitis; patent basal cisterns on CT	ELD ± craniectomy; criterion: patent basal cisterns on CT	Sustained ICP reduction is associated with reduced mortality and improved neurological outcomes
Dagod et al. (2025) [16]	Retrospective monocentric (10 years)	n = 90; severe brain injury	Well-defined ELD protocol with pre/post CT neuroimaging (Marshall score); median insertion day 6; median duration 8 days; weaning after ICP < 22 mmHg confirmed by CT	Faster ICP correction; better GOS at discharge and at 6 months vs. patients without ELD indication
Guida et al. (2024) [25]	Retrospective	n = 30; pediatric; severe TBI with ICH	ELD for ICP control in pediatric patients	ICP decreased and CPP increased post-ELD; 53% did not require further second-tier therapy after ELD
Stevens et al. (2023) [26]	Retrospective	n = 41 (ELD n = 30; EVD n = 11); TBI	ELD vs. EVD for CSF diversion	Both modalities resulted in statistically significant ICP decreases; comparable results between ELD and EVD
Chowdhury et al. (2022) [27]	Survey	36 neurosurgeons; 21 centers (UK/Ireland)	Questionnaire on the clinical practice of ELD in TBI	23% reported using ELD for refractory ICP in TBI; 56% reported use in neurovascular patients
Murad et al. (2012) [28]	Prospective	n = 15; elevated ICP	ELD placed if ICP > 20 mmHg for ≥3 h despite optimized medical management (Na > 150 mEq/L, mild hyperventilation, deep sedation, normothermia)	ICP reduced from 28.2 ± 6.5 to 10.1 ± 7.1 mmHg (p < 0.001); reduced need for hyperosmolar therapy, sedatives, and paralytics
Tömösvári et al. (2005) [29]	Interventional	n = 10; severe TBI with refractory ICP	ELD after barbiturate coma/craniectomy; management guided by BTF guidelines	Immediate and significant ICP reduction: 30.6 → 11.5 mmHg following ELD
Münch et al. (2001) [30]	Prospective Pre-post intervention	n = 23; severe brain injury/SAH-induced ischemia; patent basal cisterns	ELD after failure of hypertonic saline, barbiturate coma, and decompressive craniectomy	Notable and sustained ICP reduction with increased CPP in all patients
Mangat et al. (MTBI² study, 2025) [15]	Phase 1 RCT (protocol publication)	n = 30 (planned); severe TBI (GCS ≤ 9)	3 arms: usual care vs. early ELD (ICP ≤ 20 mmHg) vs. late ELD (ICP ≥ 20 mmHg) as first-tier 2 intervention	Primary endpoint: safety and feasibility. Secondary: identification of patient phenotype with greatest benefit (ongoing)

EVD and ELD address different aspects of intracranial hypertension and should be considered complementary interventions (Table 3). EVD is the standard of care for acute obstructive hydrocephalus and intraventricular hemorrhage, allowing simultaneous ICP monitoring [17]. ELD, inserted through a lumbar puncture, can be used in selected cases [7,12]. However, despite the limited available evidence, current management is largely guided by ICP targets [2]. Neither of the two approaches replaces the other. Their combination - instituted early - may help avoid progression to tier 3 therapies (barbiturate coma, craniectomy), which carry substantially greater morbidity [2,13-15].​​​

**Table 3:** Comparison of external ventricular drain (EVD) and external lumbar drain (ELD). ICP: intracranial pressure (mmHg); aSAH: aneurysmal subarachnoid hemorrhage; TBI: traumatic brain injury

	EVD	ELD
Indications	Acute hydrocephalus/intraventricular hemorrhage	Refractory ICP even without hydrocephalus (diffuse aSAH, TBI)
Advantages	Enables ICP monitoring via EVD	ICP-targeted drainage
	More established evidence base for ICP reduction	Lower learning curve for placement
Disadvantages	Invasive neurosurgical technique	Modest evidence in TBI; stronger evidence in aSAH
Complications	Higher infectious and hemorrhagic complications	Generally reported lower complication rates in available series

Evidence supporting ELD in ABI

This single-center case series illustrates the real-world application of ELD across a heterogeneous group of patients with refractory intracranial hypertension. Given the small sample and retrospective design, our observations are hypothesis-generating rather than confirmatory; nevertheless, several recurring themes emerge that align with the broader literature.

For the purposes of this discussion, we define early ELD placement as insertion within 48-72 hours of ICP elevation onset or ABI diagnosis, consistent with the timeframe used in prior series [14,16]. By this definition, three of our five patients (Cases 1, 2, and 3) received early placement; Case 4, in whom ELD was inserted on day 8 after failure of decompressive craniectomy, represents delayed, rescue-indication placement, in keeping with its Grade 5 CSF reserve classification. Case 5 similarly represented a late ELD placement (D6), justified by a Grade 3-4 CSF reserve classification supporting a higher ICP treatment threshold (15-22 mmHg).

In this small, heterogeneous series, early placement in a favorable anatomical substrate was descriptively associated with more favorable outcomes, although this observation should be interpreted as hypothesis-generating rather than confirmatory. The most favorable result in our series (Case 3: ELD on day 2, CSF reserve Grade 1-2, GOS 4) combined three features - early drainage, preserved CSF reserve, and lower anatomical severity. Notably, earlier timing alone was not sufficient: Case 1 received ELD on day 1 but died, reflecting overwhelming primary injury (GCS 4, Grade 1-2 imaging notwithstanding). This distinction matters: the benefit of early ELD appears contingent on an appropriate substrate rather than on timing in isolation. Our experience is consistent with reports by Soltész et al. [14] and Abadal-Centellas et al. [7], in which early drainage reduced the need for tier 2 and tier 3 escalations.

Neuroimaging-based patient selection is essential. The CSF reserve grading system proposed by Dagod et al. [16] proved clinically useful in this series. Patients with patent basal cisterns and preserved CSF reserve (Grade 1-2) are the most suitable candidates for early ELD, whereas a focal lesion with midline shift > 5 mm (Grade 5), or loss of supra- and infratentorial reserve (Grade 6-7), represents a high-risk or contraindicated scenario. Case 2 (Grade 7) illustrates that the Grade 6-7 designation of "absolute contraindication" reflects the highest-risk anatomical substrate for herniation rather than an inviolable procedural rule; in this patient, ELD was used off-label as a measure of last resort after exhaustion of all other options, accepting an elevated herniation risk in a clinical context with no remaining alternatives.

It is important to distinguish controlled, ICP-targeted lumbar drainage from the rapid, uncontrolled drainage historically associated with tonsillar herniation and increased mortality. Early reports of catastrophic coning involved free-flowing, gravity-dependent drainage without volume or rate limitation. In contrast, the studies reviewed here consistently used controlled protocols, typically limiting drainage to 5-10 mL/h (up to a maximum of approximately 15 mL/h in rescue scenarios), combined with continuous ICP monitoring and neuroimaging-based patient selection (patent basal cisterns, absence of significant mass effect or midline shift). Within these parameters, controlled ELD has been reported as safe and effective, with low rates of herniation or infectious complications [7,14,17]. In our series, drainage rates ranged from 5 mL/h (Cases 1 and 5) to 10 mL/h (Case 4), with drainage escalation guided by ICP response and imaging-based CSF reserve grade rather than fixed volume targets alone. Over-drainage was a real but manageable complication in this series: one case (Case 3) developed two episodes of over-drainage due to accidental system disconnection, requiring ELD withdrawal after one day, with no lasting clinical consequences for the patient.

Severity of primary injury remains the dominant determinant of outcome. The three cases admitted with GCS < 8 (Cases 1, 2, and 4) died despite multimodal management, including ELD. This underscores that ELD cannot rescue irreversible primary brain injury; it is most effective in patients with a degree of preserved cerebral reserve. Although the small sample size precludes formal statistical comparison, a descriptive pattern was also evident when considering global severity of illness: the three patients who died had a mean SAPS 3 of 66.3 and mean SOFA of 11.3 at admission, compared with a mean SAPS 3 of 57.0 and mean SOFA of 7.5 among the two survivors. While not conclusive given n = 5, this pattern is consistent with the broader literature indicating that admission physiological severity, rather than timing of ELD or CSF reserve grade alone, is a key determinant of outcome in critically ill neurointensive care patients [2,21]. This observation should be interpreted with caution and not as evidence of a formal severity threshold, but it reinforces that ELD outcomes in this series were shaped as much by baseline illness severity as by procedural factors.

These observations align with the broader literature. Dagod et al. reported that ELD was associated with faster ICP normalization and better GOS at discharge and at six months in a retrospective 10-year cohort [16]. Recent advances in the understanding of glymphatic physiology provide a potential mechanistic explanation for the effects of ELD beyond CSF diversion alone. By reducing pressure within the spinal CSF compartment, lumbar drainage may facilitate the movement of interstitial fluid through perivascular pathways, contributing to cerebral edema resolution and ICP control. While this mechanism remains incompletely understood, it may partly explain the sustained ICP reduction observed in some patients of this series, particularly those with preserved CSF reserve and favorable neuroimaging characteristics. Conversely, the meta-analysis by Badhiwala et al. found only weak evidence for sustained ICP reduction beyond 24 hours and no confirmed benefit on long-term outcome, partly reflecting methodological heterogeneity across included studies [13]. The ongoing MTBI² randomized trial is expected to provide higher-quality evidence on early ELD in patients with severe TBI at the tier 2 stage [15].

This case series has several important limitations that must be acknowledged. First, the retrospective, single-center design and the small sample size of five patients preclude any causal inference and limit the generalizability of our findings. The heterogeneity of the study population - encompassing patients with different etiologies, injury severities, and stages of treatment escalation at the time of ELD placement - further complicates direct comparison between cases. The absence of a control group makes it impossible to isolate the effect of ELD from other concurrent therapeutic interventions. Critically, ICP was recorded retrospectively as the highest sustained value considered clinically relevant each day, rather than through standardized, predefined-interval measurement; CPP and daily CSF drainage volume were not systematically captured for all patients. This precludes objective quantification of the physiological effect of ELD (e.g., standardized pre-/post-ELD ICP values and ICP burden above treatment threshold) and should be regarded as a methodological limitation distinct from, and compounding, the small sample size. Additionally, the lack of a standardized drainage protocol across cases - ranging from strictly ICP-triggered intermittent drainage to continuous fixed-rate drainage at 5 and 10 mL/h, individualized according to clinical response - introduces variability that precludes attributing outcome differences to drainage strategy specifically. Furthermore, the CSF reserve grading system used to classify all five patients is derived from a single, recently published monocentric cohort study and has not yet undergone independent external validation [16]; as this framework underpinned the interpretation of drainage timing across the entire series, our conclusions should be considered preliminary until this tool is validated in larger, independent cohorts. Finally, neurological outcome assessment was limited to the acute setting, without systematic long-term follow-up, restricting conclusions regarding functional recovery. Future prospective studies of ELD in this population - including the ongoing MTBI² trial [15] - should incorporate standardized, predefined-interval ICP and CPP recording alongside systematic daily drainage volume documentation to allow objective, reproducible quantification of ELD's physiological effect. These limitations underscore the hypothesis-generating nature of this report and reinforce the need for prospective, multicenter, randomized trials to establish definitive evidence on the safety, efficacy, and optimal use of ELD in refractory intracranial hypertension.

Principles proposed for clinical practice

Based on the literature and this case series, the following principles are proposed for clinical practice.

Patient Selection and Safety

Appropriate patient selection, guided by neuroimaging (patent basal cisterns, absence of significant mass effect or herniation, and CSF reserve Grade 1-4), is crucial for patient safety, an approach supported by the CT-based grading framework proposed by Dagod and colleagues [16] and by case series demonstrating that sustained ICP reduction with patent basal cisterns is associated with improved neurological outcomes [17]. Continuous neuromonitoring and pre- and post-procedure neuroimaging remain essential throughout treatment to detect early signs of over-drainage or clinical deterioration.

Timing

Early ELD placement, before tier 2 or tier 3 escalation, appears to be associated with better outcomes in less severely injured patients, a pattern consistent with reports showing significantly reduced need for tier 2/3 interventions and lower in-hospital mortality when drainage is instituted early [7,14]. This is reinforced by pediatric experience in which over half of patients required no further second-tier therapy after ELD [25].

Role Relative to Other Therapies

ELD should be considered complementary, not an alternative, to EVD and other stepwise therapies within established management algorithms such as SIBICC [2]. Comparative data suggest both modalities produce statistically significant ICP reductions, with comparable effectiveness between them [26].

Potential to Defer More Invasive Escalation

Prior interventional studies have documented immediate and sustained ICP reduction after ELD in patients with otherwise refractory hypertension [28-30], and some authors have proposed this may help reduce the need for tier 3 escalation when ELD is combined with early institution and rigorous monitoring [7,14,16]. However, this remains an unconfirmed hypothesis rather than an established clinical benefit and should not be interpreted as support for delaying decompressive craniectomy in patients who meet criteria for this time-sensitive intervention. Our own series, being small, uncontrolled, and retrospective, cannot independently support or refute this possibility, and we emphasize that ELD should be regarded as complementary to, not a substitute or gatekeeper for, established stepwise therapies including decompressive craniectomy.

Future Research

Future prospective multicenter trials - such as the ongoing MTBI² study - are critical to define optimal indications, timing, drainage protocols, and the safety profile of ELD in TBI, and to resolve the current heterogeneity in evidence regarding long-term outcome.

## Conclusions

ELD is a feasible and minimally invasive therapeutic option for refractory intracranial hypertension in ABI. The existing body of evidence - while still limited - consistently demonstrates short-term ICP reduction across multiple retrospective and prospective series, and our case series adds to this growing body of real-world experience. Evidence for improved long-term neurological outcomes and survival is more heterogeneous: while several studies report reduced mortality and better functional outcomes with early, well-selected ELD, other work - including a formal meta-analysis - found only weak evidence for sustained ICP control beyond 24 hours and no confirmed benefit on long-term outcomes. This divergence underscores the need for confirmation through prospective randomized trials.
